# Research on target detection for autonomous driving based on ECS-spiking neural networks

**DOI:** 10.1038/s41598-025-97913-4

**Published:** 2025-04-21

**Authors:** Miao Jin, Xiaohong Wang, Ce Guo, Shufan Yang

**Affiliations:** 1 School of Artificial Intelligence and Software, LiaoNing Petrochemical University, Fushun, Liaoning China; 2 Department of Neurosurgery, The Second Hospital of Shandong University, Jinan, Shandong China

**Keywords:** Computer science, Information technology

## Abstract

In response to the increasing demands for improved model performance and reduced energy consumption in object detection tasks relevant to autonomous driving, this research presents an advanced YOLO model, designated as ECSLIF-YOLO, which is based on the Leaky Integrate-and-Fire with Extracellular Space (ECS-LIF) framework. The primary aim of this model is to tackle the issues associated with the high energy consumption of traditional artificial neural networks (ANNs) and the suboptimal performance of existing spiking neural networks (SNNs). Empirical findings demonstrate that ECSLIF-YOLO achieves a peak mean Average Precision (mAP) of 0.917 on the BDD100K and KITTI datasets, thereby aligning with the accuracy levels of conventional ANNs while exceeding the performance of current direct-training SNN approaches without incurring additional energy costs. These findings suggest that ECSLIF-YOLO is particularly well-suited to assist the development of efficient and reliable systems for autonomous driving.

## Introduction

The swift progression of autonomous driving technology has underscored the pivotal importance of target detection in the decision-making processes related to this field^1^. Two primary, interconnected challenges are achieving superior performance and energy efficiency. Accurate object detection models are crucial for ensuring safe and reliable driving experiences; however, limited onboard computing resources and energy supplies necessitate reduced computational complexity and power consumption. Conventional deep learning models, such as the YOLO and RCNN series, demonstrate strong capabilities in object detection^2–4^ but demand substantial computational resources and energy, which restricts their practical application in real-world vehicles^5^.

Spiking Neural Networks (SNNs), representing the third generation of neural networks, provide a biomimetic computing framework characterized by enhanced energy efficiency, positioning them as a promising alternative for effective target detection in autonomous driving contexts^6–8^. By emulating the brain’s information processing mechanisms, SNNs can execute complex computations with lower energy expenditures and exhibit distinct advantages in managing dynamic and uncertain environments^9,10^. This methodology not only diminishes the energy consumption of onboard systems but also preserves elevated levels of detection accuracy and response speed, thereby creating new avenues for the advancement of autonomous driving technologies.

In recent years, researchers have started to investigate the applications of SNNs within the realm of autonomous driving, yielding preliminary findings. Notably, Spiking-YOLO^11^, introduced in 2020, was the inaugural model to leverage deep SNNs for object detection, establishing a foundation for subsequent research endeavors. Furthermore, EMS-YOLO^12^, released in 2023, utilizes direct-training techniques to improve SNN performance on intricate visual tasks, representing a significant advancement in the field. Despite the inherent advantages of SNNs in terms of energy efficiency, they still fall short of traditional ANN in overall performance, indicating a potential area for further enhancement. Addressing this performance gap could significantly accelerate the evolution of autonomous driving technologies.

In response to the current state of research, we introduce a novel spiking neuron model known as ECS-LIF. The primary differentiation of the ECS-LIF model from the traditional LIF model is its forceful regulatory mechanism, which incorporates the intricately integrated microenvironment of brain cells, specifically the extracellular space (ECS).

While the conventional LIF model focuses exclusively on simulating the processes of membrane potential integration and spike generation within neurons^13^, the ECS-LIF model encompasses both a temporal domain model-capturing the effects of neuronal firing on the ECS and its self-recovery characteristics-and a spatial domain model-utilizing convolution operations to represent the diffusion effects of signaling molecules within the ECS. This model uniquely integrates the bidirectional interactions between neurons and the ECS into its computational framework, thereby emulating the adaptive features of biological nervous systems. Consequently, this approach significantly enhances the biological interpretability of pulsed neural networks, improves their classification accuracy and robustness, and offers a more realistic and comprehensive framework for the understanding and simulation of biological nervous systems.

The ECSLIF-YOLO model developed based on this approach aims to leverage the advantages of ECS-LIF to further enhance the performance of Spiking Neural Networks (SNNs) in target detection tasks. It strives to achieve higher accuracy and real-time performance while ensuring low energy consumption. This research not only advances the theoretical and technological development of SNNs but also paves the way for new opportunities in the practical application of object detection systems for autonomous driving.

The main contributions of this paper are as follows.

- The integration of ECS-LIF neurons, along with the influence of ECS, is proposed. In comparison to standard Leaky Integrate-and-Fire (LIF) neurons, this approach more effectively simulates the behavioral characteristics of biological neurons and significantly enhances the model’s performance in complex dynamic environments.
- The ECSLIF-YOLO model, a direct training pulse neural network designed for automatic driving target detection, has been developed. This model utilizes ECS-LIF neurons, which enhance performance compared to existing direct training pulse neural networks.
- The experimental results indicate that, when evaluated on the KITTI and BDD100K datasets, the proposed model achieves performance levels that are comparable to or surpass those of artificial neural networks (ANN) and existing direct training spiking neural network (SNN) methodologies, all while maintaining energy consumption levels that are consistent with those of the current direct training SNN approaches.

## Related work

### Object detection for autonomous driving

In the domain of autonomous driving, object detection plays a pivotal role in enabling vehicles to make swift and informed decisions through the precise identification and classification of objects in real time. This process necessitates the efficient processing of substantial volumes of data. Current frameworks for object detection are primarily classified into two categories: two-stage frameworks, such as the RCNN series, and single-stage frameworks, exemplified by YOLO and the Detr series. Two-stage frameworks employ a Region Proposal Network to enhance the accuracy of localization and classification; however, this approach incurs significant computational costs, which can hinder real-time performance. Conversely, single-stage frameworks predict object locations and categories directly from images without the need for generating candidate regions, thereby facilitating faster inference and simpler architectures that are more suitable for real-time applications or environments with limited resources.

The utilization of Spiking Neural Networks (SNNs) for target detection is garnering increasing interest due to their energy efficiency and biological plausibility. SNNs emulate biological neurons by conveying information through discrete spikes^14–16^, which may lead to reduced power consumption and enhanced parallelism when implemented on neuromorphic hardware. Nevertheless, several practical challenges persist. The integration of efficient single-stage detectors, such as YOLO, into SNNs remains in its early stages, with only limited success reported. Current methodologies include the conversion of pre-trained Artificial Neural Network (ANN) models into their SNN counterparts and the direct training of YOLO variants as SNNs, such as EMS-YOLO. Although these approaches demonstrate potential, they have yet to achieve the performance levels of traditional ANNs, particularly in the context of high-resolution images or complex scenes, where both detection accuracy and speed may not meet practical requirements. Therefore, while SNNs present promising avenues for advancing object detection efficiency, further developments are necessary to address existing limitations.

### Spiking neurons

Spiking Neurons are computational models in neuroscience and neuromorphic engineering designed to simulate the behavior of neurons in biological nervous systems more realistically. Unlike traditional artificial neural networks (ANNs) based on continuous activation functions, spiking neurons transmit information through electrical pulses or ”spikes” at discrete time points. This time-based communication not only better reflects brain function but also offers unique advantages in processing dynamic data, pattern recognition, and learning tasks^17^.

Sparse coding is a significant feature of spiking neurons, characterized by sparse pulse activity that reduces unnecessary computational burden and energy consumption, thereby improving energy efficiency, particularly in large-scale networks^18^. Additionally, spiking neurons possess complex nonlinear dynamic properties, including membrane potential accumulation and leakage and post-discharge resetting, enabling them to perform more sophisticated information processing tasks than traditional artificial neurons. Synaptic plasticity mechanisms, such as spike-timing-dependent plasticity (STDP), allow for adjusting connection weights based on input signals, enhancing performance and adaptability^19^.

The simplest spiking neuron model is the Integrate-and-Fire (IF) model, which assumes neurons accumulate input until reaching a threshold, then fire a spike and reset. The Leaky Integrate-and-Fire (LIF) model extends this concept by incorporating a leakage property, allowing the membrane potential to gradually decay back to a resting state without sufficient stimulation^20^. More complex models, such as the Izhikevich and Hodgkin-Huxley models, account for detailed ion channel dynamics to more accurately simulate various types of real neuron behavior.

### EMS-YOLO model

EMS-YOLO is an innovative framework designed for efficient object detection. Its core component, the Energy-efficient Membrane Shortcut ResNet (EMS-ResNet), introduces a novel direct training method for Spiking Neural Networks (SNNs). EMS-ResNet employs a fully spiking residual block structure and integrates the LIF neuron model before each convolution operation in the residual path. This design enables information transmission in the form of sparse spikes along the main data flow path, while information on the residual path is converted into similarly sparse spike signals. Consequently, the entire network operates within the spiking domain, significantly reducing the need for additional floating-point operations.

This innovative design enhances the depth of SNNs that can be directly trained while maintaining low energy consumption throughout network operation. Even as network architecture becomes more complex and deeper, the framework sustains high detection performance while significantly reducing energy consumption. Such advancements are crucial for high-performance, low-power applications, enabling more energy-efficient target detection solutions without compromising detection accuracy.

Moreover, EMS-YOLO’s integration of LIF neurons facilitates more biologically realistic simulations, enhancing the model’s robustness and adaptability in dynamic and uncertain environments. This high-efficiency, low-power nature makes EMS-YOLO ideal for real-time applications such as autonomous driving, where the system must respond quickly and operate stably over extended periods without overheating or excessive battery drain.

## Method

### The ECS-LIF model

The Extracellular Space (ECS) is a sophisticated domain surrounding neurons, playing an essential role in neural signaling. Recent research has highlighted that the ECS influences not only the communication between neurons but also stands at the heart of higher brain functions, including emotion, memory formation, cognitive abilities, and the pathological mechanisms underlying mental illnesses^21–23^. Despite the increasing recognition of the ECS’s significance, most current spiking neural networks (SNNs), such as the Leaky Integrate-and-Fire (LIF) model, concentrate mainly on simulating the dynamics of neurons themselves, largely overlooking the impacts of the surrounding microenvironment.

To more accurately reflect the true workings of the biological nervous system, our team developed the ECS-Leaky Integration-and-Fire (ECS-LIF) model, which integrates the ECS mechanism into the LIF framework. The model consists of two parts, time domain model and space domain model, which simulate the temporal dynamic characteristics of neurons and ECS and the spatial diffusion effect of signals in ECS respectively.

#### Time domain model

The time-domain model characterizes the effects of neuronal firing on the microenvironment of brain cells, specifically the ECS, as well as the feedback mechanisms through which the ECS influences neurons. These interactions include self-recovery processes, ECS stimulation by neurons, and ECS-mediated responses affecting neuronal membrane potentials.

The ECS state of the *i*-th neuron at time *t* is defined as $ECS^{i} \left( t \right)$, and its dynamic characteristics include the following mechanisms:

1. Self-Recovery: In the absence of neuronal stimulation over some time, the ECS state gradually reverts to its initial condition. This process is mathematically represented by the following equation:

1 $$\begin{aligned}&ECS^{i} \left( t \right) \sim \left( 1-\frac{1}{\tau _{ECS} } \right) ECS^{i} \left( t-1 \right) . \end{aligned}$$

Where, $\tau _{ECS}$ is the time constant describing the recovery of ECS, which is used to constrain the speed of recovery..

2. ECS Stimulation by neurons: When neurons are activated at a specific moment, they release spiking signal $S^{i} \left( t \right)$ that update the ECS state. The updating mechanism is described by the following formula:

2 $$\begin{aligned} ECS^{i} \left( t \right) = \left( 1-\frac{1}{\tau _{ECS} } \right) ECS^{i} \left( t-1 \right) + \gamma S^{i} \left( t \right) . \end{aligned}$$

where $\gamma$ describes how strongly the ECS responds to neuronal excitation, with higher values signifying a more pronounced response.

3. ECS Reaction on neurons: The updated ECS state exerts a feedback effect on neurons, influencing changes in their membrane potentials. A saturation function $f \left( \cdot \right)$ is employed to constrain this interaction. Assuming the ECS effect on the i-th neuron is denoted as $M^{i} \left( t \right)$:

3 $$\begin{aligned}&M^{i} \left( t \right) = \beta f\left( ECS^{i} \left( t \right) \right) . \end{aligned}$$

Where $\beta$ is the maximum influence degree of ECS, the saturation function of this work is selected:

4 $$\begin{aligned}&f\left( x \right) = tanh\left( x \right) . \end{aligned}$$

#### Spatial domain model

In biological neural networks, apart from direct linear transmission of neural signals via synapses, there exists a volume transmission mode mediated by ECS diffusion, forming the spatial domain model of ECS. This model simulates the diffusion dynamics of neural signals within the ECS:

1. Convolutional layer diffusion: Given that ECS diffusion in this layer can be conceptualized as the influence of neighboring ECS components on other neurons in the network, convolution operations are utilized to simulate this diffusion effect. Convolutional kernel weights are randomly initialized to mimic the intricate structure of biological ECS. Each iteration of ECS updates involves a convolution operation to simulate the diffusion effect after weight adjustments. The updating formula is provided below:

5 $$\begin{aligned}&ECS^{i} \left( t \right) = \left( 1-\frac{1}{\tau _{ECS} } \right) ECS^{i} \left( t-1 \right) + \gamma \left( Conv\left( S^{i}\left( t \right) ,W_{conv} \right) +b_{conv} \right) . \end{aligned}$$

where $W_{conv}$ and $b_{conv}$ are the weight and bias of the convolution kernel, respectively, and the diffusion path can be optimized through training.

2. Fully connected layer diffusion: In fully connected layers, where neurons are interconnected, ECS diffusion spreads across all neurons in the layer. Linear transformations using unit matrix weights $W_{linear}$ and zero bias $b_{linear}$ are employed to simulate this phenomenon:

6 $$\begin{aligned}&ECS^{i} \left( t \right) = \left( 1-\frac{1}{\tau _{ECS} } \right) ECS^{i} \left( t-1 \right) + \gamma \left( S^{i}\left( t \right) \cdot W_{linear}+b_{linear} \right) . \end{aligned}$$

where, $$W_{linear} = I(UM5)$$, $$b_{linear}=0$$

#### ECS-LIF model

The time-domain and spatial-domain models are integrated into the LIF neuron framework.

Equation (7) describes the membrane potential renewal formula for the ECS-LIF model:

7 $$\begin{aligned}&V_{i}^{t+1,n+1} = \tau V_{i}^{t,n+1} \left( 1 - X_{i}^{t,n+1} \right) + {\textstyle \sum _{j} } W_{ij}^{n} X_{j}^{t+1,n} + \beta \cdot f \left( ECS_{i}^{t,n+1} \right) . \end{aligned}$$

where, $V_{i}^{t,n+1}$ is the membrane potential of the *i*-th neuron in layer $n+1$ at time step *t*, and $\tau$ is the leaking decay factor. The input to a synapse is the sum of *j* spikes with synaptic weights from the previous layer *n*. $\beta \cdot f \left( ECS_{i}^{t,n+1} \right)$ is the influence of the microenvironment of the *i* neuron in layer $n+1$ on it at time step *t*, $\beta$ is the maximum influence of ECS, and $ECS_{i}^{t,n+1}$ is the ECS state at that time.Once the neuron spikes at time $t+1$, its membrane potential $V ^{t+1,,n+1}$ is reset to a reset potential $V_{reset}$.

Equation (8) outlines the spike release formula:

8 $$\begin{aligned}&X_{i}^{t+1,n+1} = H\left( V_{i}^{t+1,n+1} - V_{th} \right) . \end{aligned}$$

Where, $H\left( \cdot \right)$ represents the step function, when$x \ge 0$, $H\left( x \right) =1$, otherwise $H\left( x \right) =0$.

Equation (9) specifies the ECS renewal formula:

9 $$\begin{aligned}&ECS_{i}^{t+1,n+1}=\left\{ \begin{array}{l}\left( 1-\frac{1}{\tau _{ECS}}\right) ECS_{i}^{t,n+1}+\gamma \left( \operatorname {Conv}\left( X_{i}^{t,n+1}, W_{conv}\right) +b_{conv}\right) \text{ Convolution } \text{ layer } \\ \left( 1-\frac{1}{\tau _{ECS}}\right) ECS_{i}^{t,n+1}+\gamma \left( X_{i}^{t,n+1} \cdot W_{linear}+b_{linear}\right) \text{ Fully } \text{ connected } \text{ layer }\end{array}\right. \end{aligned}$$

Where, $\tau _{ECS}$ represents the ECS decay factor, $\gamma$ describes the intensity of the ECS response to neuronal excitation.

### Training strategy

In the training process of Spiking Neural Networks (SNNs), the conventional backpropagation algorithm cannot be directly applied due to the non-differentiability of spike signals. To address this challenge, we implement the Surrogate Gradient^24^ Method by introducing a differentiable approximation function to substitute for the Heaviside step function, thereby enabling efficient error propagation across layers.Specifically, at each time step *t* of neuron *i*, the derivative of its output $X_{i}^{t,n}$ with respect to the membrane potential $V_{i}^{t,n}$ can be expressed as:

10 $$\begin{aligned} \frac{\partial X_{i}^{t,n} }{\partial V_{i}^{t,n} } =\frac{1}{a} sign\left( \left| V_{i}^{t,n} - V_{th} \right| \le a \right) . \end{aligned}$$

Where *a* is a parameter that ensures that the integral gradient is 1 and determines the steepness of the curve.

In addition, to further improve the performance of the model, we use the threshold-dependent batch normalization (TDBN)^25^ of the time domain under consideration. TDBN not only considers the changes in the spatial dimension but also optimizes the time series data, thus effectively solving the problems of instability and efficiency in the SNN training process. Specifically, TDBN can normalize the input $i_{i}^{t+1}$ with the following formula:

11 $$\begin{aligned}&TDBN\left( I_{i}^{t+1}\right) = \lambda _{i}\frac{\alpha V_{th}\left( I_{i}^{t+1} - \mu _{i}^{2}\right) }{\sqrt{\sigma _{i}^{2}+\epsilon } } +\beta _{i}. \end{aligned}$$

Among them, the $\mu _{i}^{2}$ and $\sigma _{i}^{2}$ are the mean and variation values for every channel using a mini-batch of sequential inputs $\left\{ I_{i}^{t+1}= {\textstyle \sum _{j} W_{ij}^{t+1,n} +\beta \cdot f\left( ECS_{i}^{t,n+1} \right) } |t=0, \cdots ,T+1 \right\}$, $\epsilon$ is a tiny constant to avoid dividing by zero, $\lambda _{i}, \beta _{i}$ are two trainable parameters, and $\alpha$ is a threshold-dependent hyper-parameter.

By combining the proxy gradient method and the TDBN normalization strategy, the model not only overcomes the challenge of non-differentiable pulse signal but also improves the training efficiency and performance of SNN.

### The ECSLIF-YOLO model

The input data can be expressed as $\textbf{X}=\left\{ X_{t} \right\} _{t=1}^{T}$, where the dimension of the input data $X_t$ for each time step *t* is $C\times H\times W$, *C* is the number of channels, and $H\times W$ correspond to the resolution of the height and width, respectively.To achieve this, we calculate the information of *N* objects $\textbf{B} =\left\{ B _{n} \right\} _{n=1}^{N}$by:

12 $$\begin{aligned}&\textbf{B} =\mathscr {D} \left( \textbf{X} \right) . \end{aligned}$$

**Fig. 1:**
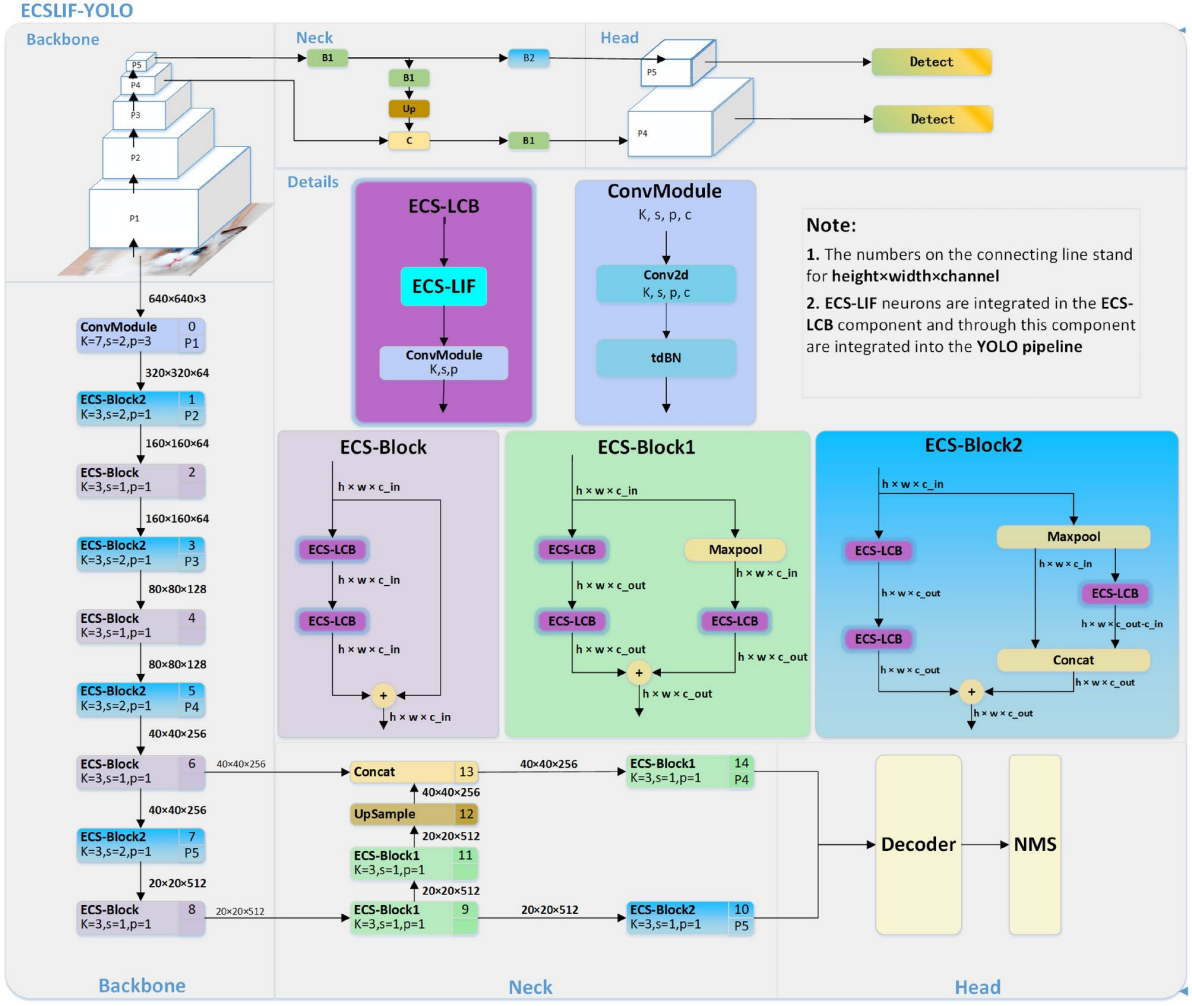
The ECSLIF-YOLO (ResNet18) structure diagram.

Where the $B _{n} =\left\{ x_{n}, y_{n},w_{n},h_{n},c_{n},f_{n} \right\}$ is the bounding box of every forecast object position and categories. The $\left( x_{n}, y_{n} \right) ,w_{n},h_{n}$ denotes the coordinates of the top-left corner, width, and height of the bounding box, respectively.The parameters $c_{n}$ and $f_{n}$ respectively refer to the object category and confidence score.$\mathscr {D}$ refers to the ECSLIF-YOLO model, whose structure diagram is shown in Fig. 1.

ECSLIF-YOLO is an advanced spiking neural network (SNN) object detection model that integrates ECS properties and LIF neuron models to create a distinctive ECS-LIF neuron structure, thereby enhancing performance and energy efficiency in object detection tasks. The model comprises three main components: the backbone network for feature extraction, the neck network for feature enhancement, and the head module for object detection. The ECS-Block serves as the core unit of the model, encompassing the ECS-LCB and residual connection paths. Additionally, the ECS-LCB is further divided into ECS-LIF neurons, convolution operations, and batch normalization steps.

The backbone network, responsible for feature extraction, adopts an architecture akin to ResNet with adaptive modifications. The initial convolutional layer automatically converts input data into spike format, eliminating the need for additional encoding procedures. ECS-LIF neurons integrate weighted input signals, generating spike trains when the membrane potential reaches a threshold value. Target features are extracted through a series of ECS-blocks with varying dimensions and channel numbers, ultimately passing the obtained features forward.

The neck network consists of multiple ECS-Blocks, each containing ECS-LCB units (comprising ECS-LIF neurons, convolution operations, and batch normalization) and residual connection paths. This design minimizes SNN performance degradation while improving information flow transmission efficiency, ensuring efficient feature propagation from the backbone to the head module.

The detection head is tasked with transmitting the most recent membrane potential of the neuron^26^ to each detector, facilitating the generation of anchors of varying dimensions. Following the application of Non-Maximum Suppression (NMS), the definitive classifications and bounding box coordinates for the various objects are derived. The ecslf-yolo model is trained utilizing the cross-entropy loss function.

## Results and discussion

### The dataset and experimental environment of the experiment

The data sets of this experiment used 2d data sets of BDD100K and KITTI data sets.

The BDD100K dataset, published by the University of Berkeley AI Lab (BAIR), is one of the largest and most diverse publicly available driving datasets, containing 70,000 training images, 10,000 validation images, and 20,000 test images. The original categories include cars, buses, pedestrians, bicycles, trucks, motorcycles, trains, riders, traffic signs, and traffic lights. For a more detailed classification, this paper further categorizes traffic lights into four types: red, green, yellow, and no light, thereby expanding the total number of categories to thirteen.

The KITTI dataset, jointly created by Karlsruhe Institute of Technology and Toyota American Institute of Technology, is currently the world’s largest computer vision algorithm evaluation dataset for autonomous driving scenarios, containing 7481 training images and 7518 test images. The original categories were cars, vans, trucks, trams, pedestrians, seated people, cyclists and miscellaneous. To simplify the process, this paper combines pedestrians and seated people into one category and eliminates miscellaneous categories, leaving six main categories.

Experimental setup: To simplify the mathematical model and make the updating of state variables more intuitive, we set the reset value of the LIF neuron $V_{reset}=0$. To enhance the sensitivity of neurons to changes in input signals and enable them to process rapidly varying information (e.g., dynamic environments in autonomous driving scenarios), we set the membrane time constant $\tau =0.25$. The threshold $V_{th}$ is set to 0.5 to ensure that neurons fire at an appropriate input intensity, avoiding both excessive sensitivity and insensitivity. Additionally, the coefficient $\alpha$ is set to 1, preserving the original ratio of the input signal.

For ECS-LIF neurons, building upon the aforementioned settings, we further define the ECS attenuation constant $\tau _{ECS}=5.0$. This parameter simulates the prolonged effects of extracellular substance concentration changes, enabling the model to capture dynamics on a longer timescale. The intensity of ECS response to neuronal excitation $\gamma$ is set to 0.75, ensuring a moderate yet not overly strong reaction. Furthermore, the maximum ECS influence degree $\beta$ is set to 0.25, limiting the ECS impact on neurons even under extreme conditions. This ensures overall model balance and preserves the significance of other factors, such as direct synaptic connections.

The SGD (Stochastic Gradient Descent) optimizer is employed for weight updates, with the learning rate set to $1e ^{-2}$, a moderate value that guarantees rapid convergence while mitigating oscillation or divergence issues caused by excessively large step sizes. The model was trained for 300 epochs on the autonomous driving datasets KITTI and BDD100K, using a batch size of 16, which strikes a favorable trade-off between training speed and memory consumption. Practical training results confirmed that this configuration not only completes training within a reasonable timeframe but also maintains excellent generalization and stability.

### Experimental evaluation index

When evaluating the performance of an autonomous driving object detection model, a series of key metrics are typically used, of which the most important are the mean accuracy (mAP), detection speed, and energy consumption. Here is a detailed explanation of these metrics:

mAP: mAP is one of the most commonly used comprehensive evaluation indicators in object detection, which measures the overall performance of a model in different categories. The two common mAP calculations are mAP@0.5 and mAP@0.5:0.95. The former is to calculate the average accuracy (AP) of each class when the IoU threshold is set to 0.5, and then average all classes, while the latter is to calculate the Maps separately on different IoU thresholds (from 0.5 to 0.95, with a step size of 0.05), and then average these mAP values.

Detection speed: Detection speed refers to the number of images that the model can process in one second, and it is an important index to measure the real-time performance of the model.

Energy consumption: Based on the theoretical neuromorphic hardware framework, the energy consumption characteristics of artificial neural network (ANN) and impulsive neural network (SNN) are compared and analyzed in this study. In ANN, every operation involves the multiplication and addition (MAC) of floating-point numbers, and the computational burden is usually quantified by the number of floating-point operations (FLOPs). In contrast, Spiking Neural Networks (SNNs) are energy-efficient when implemented on neuromorphic hardware: neurons only trigger cumulative computation (AC) when they emit pulses, without the need to continuously engage in floating-point operations. Based on this difference, the total energy consumption of SNN can be abstracted as $E_{SNN} = {\textstyle \sum _{n} } E_{b}$ for each block:

13 $$\begin{aligned} E_{b} = T \times \left( E_{AC} \times OP_{AC} + E_{MAC} \times OP_{MAC} \right) . \end{aligned}$$

Where the $T$ represents the time step, the energy consumption of each is determined by the number of AC and MAC operations($OP_{AC} , OP_{AC}$).

The energy consumption of ANNS can be abstracted as $E_{ANN} = {\textstyle \sum _{n} } E_{b}$, for each block:

14 $$\begin{aligned}&E_{b} = E_{AC} \times OP_{AC} + E_{MAC} \times OP_{MAC} . \end{aligned}$$

It is worth noting that the above model is based on theoretical derivation, assuming that the data for various operations are 32-bit floating-point implementation in 45nm technology^27^, where $E_{AC} = 6pj$ and $E_{MAC} = 1.1pj$. This model does not cover actual hardware features (such as dynamic power consumption for Intel Loihi and IBM TrueNorth chips) and environment variables. Therefore, it is necessary to further optimize the accuracy of the model through experimental verification: by deploying predefined algorithms on the target hardware platform, collecting and analyzing its dynamic energy consumption data in real time, to calibrate theoretical parameters and improve the model adaptability.

Owing to the unavailability of experimental apparatus, this research was limited to theoretical estimations regarding energy consumption and was unable to conduct empirical validation on actual neuromorphic hardware.

### Comparative analysis of experimental results

The performance of the proposed ECSLIF-YOLO model on the KITTI dataset is summarized in Table 1. This model is compared against an ANN model with an identical network architecture, the existing YOLO model, and the EMS-YOLO model to evaluate the differences in both performance and energy consumption. To ensure statistical reliability, multiple experiments were conducted, and the average values are reported. Due to the inherent energy-saving advantages of Spiking Neural Networks (SNNs) in neuromorphic hardware, where neurons participate in accumulative computation (AC) only when spikes occur, thereby avoiding floating-point operations and setting the operand $OP_{AC}$ to 0, the energy consumption of different models can be quantified using Equations (13) and (14) (Note:$1mJ=10^9pj$).

Among them, Res18 and Res10 in the ANN model are standard ResNet for fair comparison, and their network structure, number of network layers, number of channels, etc., are the same as Res18 and Res10 in ECSLIF-YOLO.

**Table 1:** mAP and energy consumption of different models on kitti dataset.

Method	Work	Models	$OP_{AC}$	$OP_{MAC}$	mAP@0.5	T	Energy
BP	ANN	Res10	37.9M	37.9M	0.893	–	0.270mj
		Res18	45.8M	45.8M	0.913	–	0.325mj
	YOLOv8	n	3.27M	3.27M	0.845	–	0.023mj
		s	11.6M	11.6M	0.906	–	0.082mj
		m	26.6M	26.6M	0.921	–	0.189mj
	YOLOv10	n	2.7M	2.7M	0.810	–	0.019mj
		s	8.07M	8.07M	0.895	–	0.057mj
		m	16.5M	16.5M	0.907	–	0.117mj
	YOLOv11	n	2.59M	2.59M	0.826	–	0.018mj
		s	9.43M	9.43M	0.894	–	0.067mj
		m	20.0M	20.0M	0.924	–	0.142mj
Directly-Trained SNN	EMS-YOLO	Res10	2048	0	0.888	4	2252.8pj
		Res18	4096	0	0.902	4	4505.6pj
	ECSLIF-YOLO(our)	Res10	2048	0	0.895	4	2252.8pj
		Res18	4096	0	0.917	4	4505.6pj

All models utilize the SGD optimizer with a learning rate (lr0) of $1e ^{-2}$, momentum of 0.937, weight decay of 0.0005, and no pre-trained weights.

Table 1 shows that the ECSLIF-YOLO model achieves excellent detection accuracy, with mAP@0.5 values of 0.895 and 0.917 for ResNet-10 and ResNet-18, respectively, surpassing both the ANN model with the same structure and the existing SNN method (EMS-YOLO). Notably, within four time steps, the ECSLIF-YOLO model’s performance is comparable to current conventional YOLO models, such as YOLOv8-s (0.906) and YOLOv11-m (0.924). Moreover, the theoretical energy consumption of the ECSLIF-YOLO model is only 2252.8 pj and 4505.6 pj, which is significantly lower than traditional YOLO models and comparable to the EMS-YOLO model.

These results confirm that the ECSLIF-YOLO model successfully balances high-precision target detection with low energy consumption, making it particularly suitable for applications like autonomous driving, where real-time performance and energy efficiency are critical. By performing computations only at spike events, SNNs greatly reduce unnecessary floating-point operations, thereby minimizing energy consumption. Additionally, this study demonstrates that even while maintaining high performance, the ECSLIF-YOLO model exhibits a lower energy footprint than traditional YOLO models, proving that optimized SNNs can serve as ideal choices in resource-constrained environments. Future work will focus on further optimizing the model and exploring its potential for broader practical applications.

Next, we discuss the tradeoff between the accuracy, inference speed, and energy consumption of the ECSLIF-YOLO model, and the FPS of some models are shown in Table 2.

**Table 2:** FPS for some models.

Models	ANN-Res18	YOLOv8m	YOLOv10m	YOLOv11m	EMS-Res18	ECS-Res18
FPS	102.2	98.6	97.0	100.2	76.7	75.3

The experimental environment is 4060ti.

As illustrated in the table, the ECSLIF-YOLO model exhibits a slightly lower frames per second (FPS) rate of 75.3 compared to other non-ECS models, indicating a minor increase in latency. However, it still maintains the capability to process over 75 frames per second, which meets the requirements for many autonomous driving tasks. In this context, the model’s advantages in energy consumption more than compensate for the slight decrease in FPS. ECS technology can significantly reduce energy consumption without adversely impacting real-time responsiveness, making it a viable option in the field of autonomous driving.

This study examines the performance disparities between the ECS-LIF neuron model and the conventional LIF neuron model when subjected to varying intensities of Gaussian noise. In particular, Gaussian noise was introduced at the pixel level in the input data, and the experiments were conducted multiple times to enhance the reliability of the results. By assessing the mean Average Precision at a threshold of 0.5 (mAP@0.5) for both models across different noise conditions, we quantitatively analyze and compare their performance in environments characterized by noise. The results of these experiments are summarized in Table 3, which underscores the significance of the findings and includes 95% confidence intervals that reflect the performance variations between the two models at different noise intensity levels.

**Table 3:** mAP of ECS-LIF and LIF neurons under different noise intensities.

Noise intensity	Models	Mean	SD	p-value	Confidence Interval(95%)
0.2	LIF	0.803	0.0036	0.021*	(00.7920.8068)
	ECS-LIF	0.811	0.0035		(0.8730.8147)
0.4	LIF	0.610	0.0038	0.0036*	(0.6060, 0.6140)
	ECS-LIF	0.676	0.0040		(0.6718, 0.6802)
0.6	LIF	0.426	0.0035	0.0011**	(0.6718, 0.6802)
	ECS-LIF	0.498	0.0042		(0.49360.5024)
0.8	LIF	0.312	0.0041	<0.001***	(0.3077, 0.3163)
	ECS-LIF	0.391	0.0036		(0.3872, 0.3948)

The experimental setup is the same as in Subsection "The dataset and experimental environment of the experiment".

**Fig. 2:**
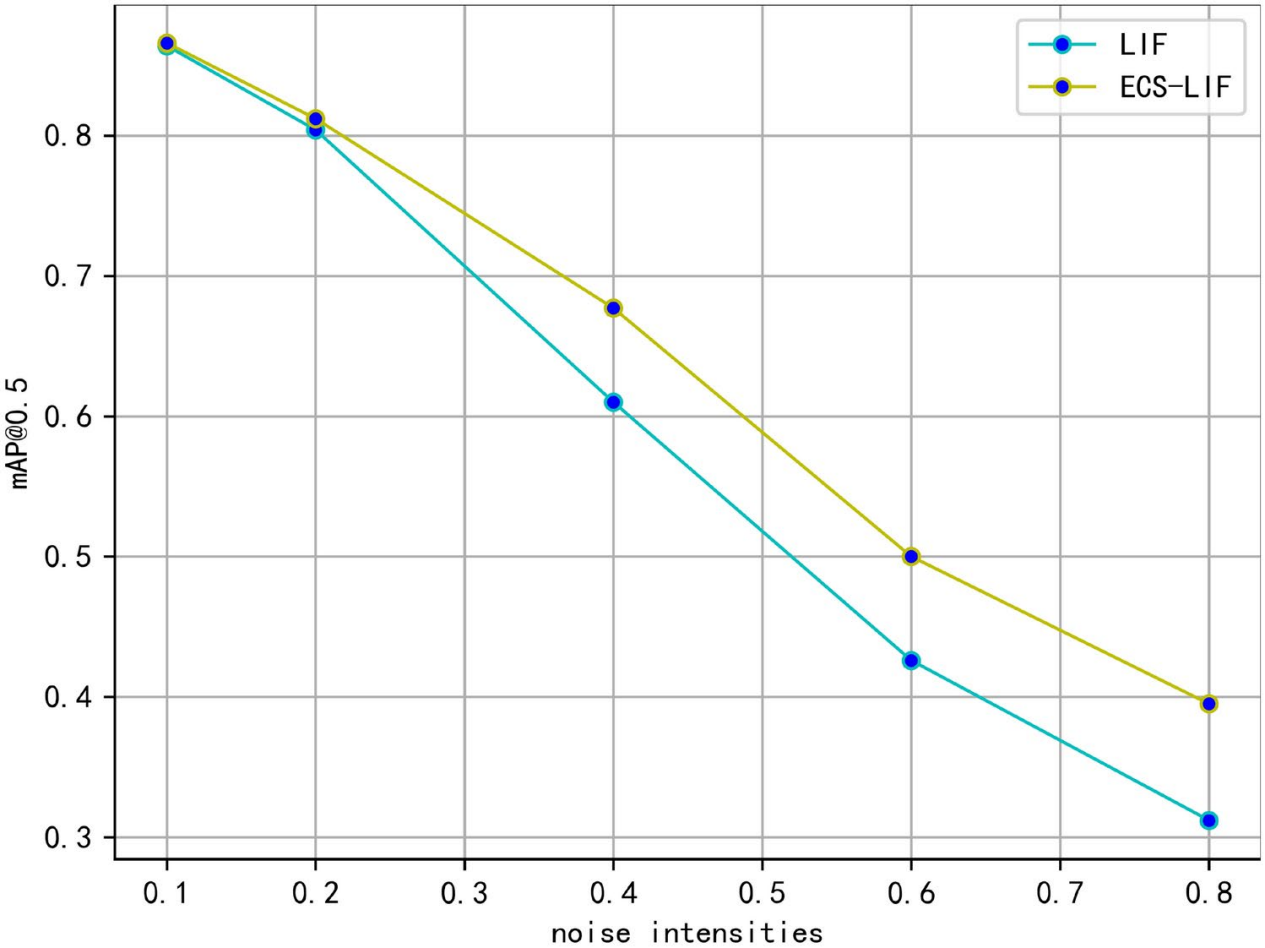
Comparison of ECS-LIF and LIF at different noise intensities.

Statistical analysis based on repeated experiments revealed that the ECS-LIF model significantly outperformed the traditional LIF model across all noise intensities ($p < 0.05$, independent sample t-test). As shown in Table 3, when noise intensity increased from 0.2 to 0.8, the accuracy of the ECS-LIF model decreased by 51.8%, significantly less than the 61.1% decrease observed in the LIF model. At the highest noise level (0.8), the mean accuracy of the ECS-LIF model was 0.391 (±0.0036), which is 25.0% higher than that of the LIF model (0.312 ± 0.0041) ($p = 5.2 \times 10^{-5}$)). This can be seen more intuitively in Fig. 2. The experimental standard deviation for both models was below 0.5%, indicating highly reproducible results.

The superior noise robustness of the ECS-LIF model is attributed to its bionic design, which deeply models the dynamic characteristics of the brain cell microenvironment (ECS). The time-domain mechanism dynamically adjusts the membrane potential threshold through the self-recovery characteristic of ECS and bidirectional feedback between neurons and ECS, ensuring reliable signal transmission. The spatial-domain mechanism simulates the local diffusion effect of ECS and global coordination via convolutional operations, dispersing the influence of single neurons’ noise interference to neighboring regions, thus achieving collaborative noise reduction at the network level.

**Fig. 3:**
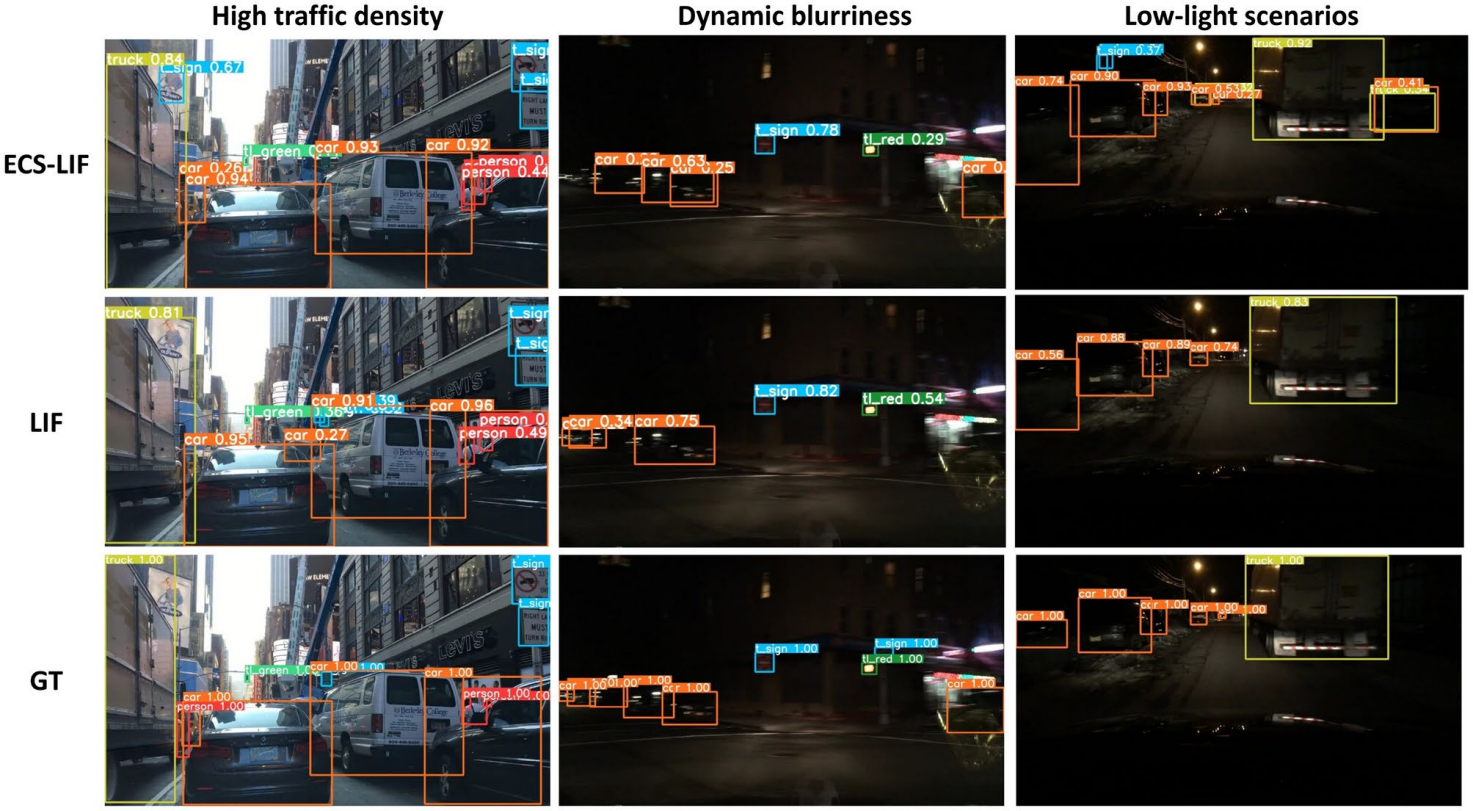
Detection results of ECS-YOLO (Res10) model on the BDD100K dataset in different time steps.

Following this, an experimental analysis was conducted to assess the robustness of ECS-LIF neurons in practical applications. The findings are presented in Fig. 3, where the time step is uniformly maintained at 3, and all other parameters remain consistent with those outlined in Subsection "The dataset and experimental environment of the experiment".

The data illustrated in Fig. 3 indicates that ECS-LIF neurons surpass the conventional LIF neuron model in scenarios characterized by High traffic density, Dynamic blurriness, and Low-light scenarios.

Specifically, in environments with High traffic density, ECS-LIF neurons display superior accuracy in the identification of vehicles and other objects, along with increased confidence in detecting boxes. In Dynamic blurriness, ECS-LIF neurons sustain a high level of recognition accuracy, whereas LIF neurons experience a rise in both false positives and missed detections. Additionally, under Low-light scenarios, ECS-LIF neurons continue to perform exceptionally well, accurately identifying targets, while the performance of LIF neurons significantly declines. These results imply that ECS-LIF neurons exhibit enhanced robustness and adaptability in complex environments, highlighting their potential for practical applications.

To assess the impact of time step on the performance of the proposed autonomous driving target detection model, we conducted supplementary experimental analyses, the results of which are detailed in Table 4.

**Table 4:** mAP of object detection for the ECSLIF-YOLO (Res10) model on the BDD100K dataset at various time intervals.

Work	T	1	2	3
ECSLIF-YOLO(my)	mAP@0.5	0.193	0.217	0.230
	mAP@0.5:0.95	0.083	0.095	0.100
EMS-YOLO	mAP@0.5	0.193	0.198	0.206
	mAP@0.5:0.95	0.083	0.085	0.091

The experimental setup is the same as in Subsection "The dataset and experimental environment of the experiment".

**Fig. 4:**
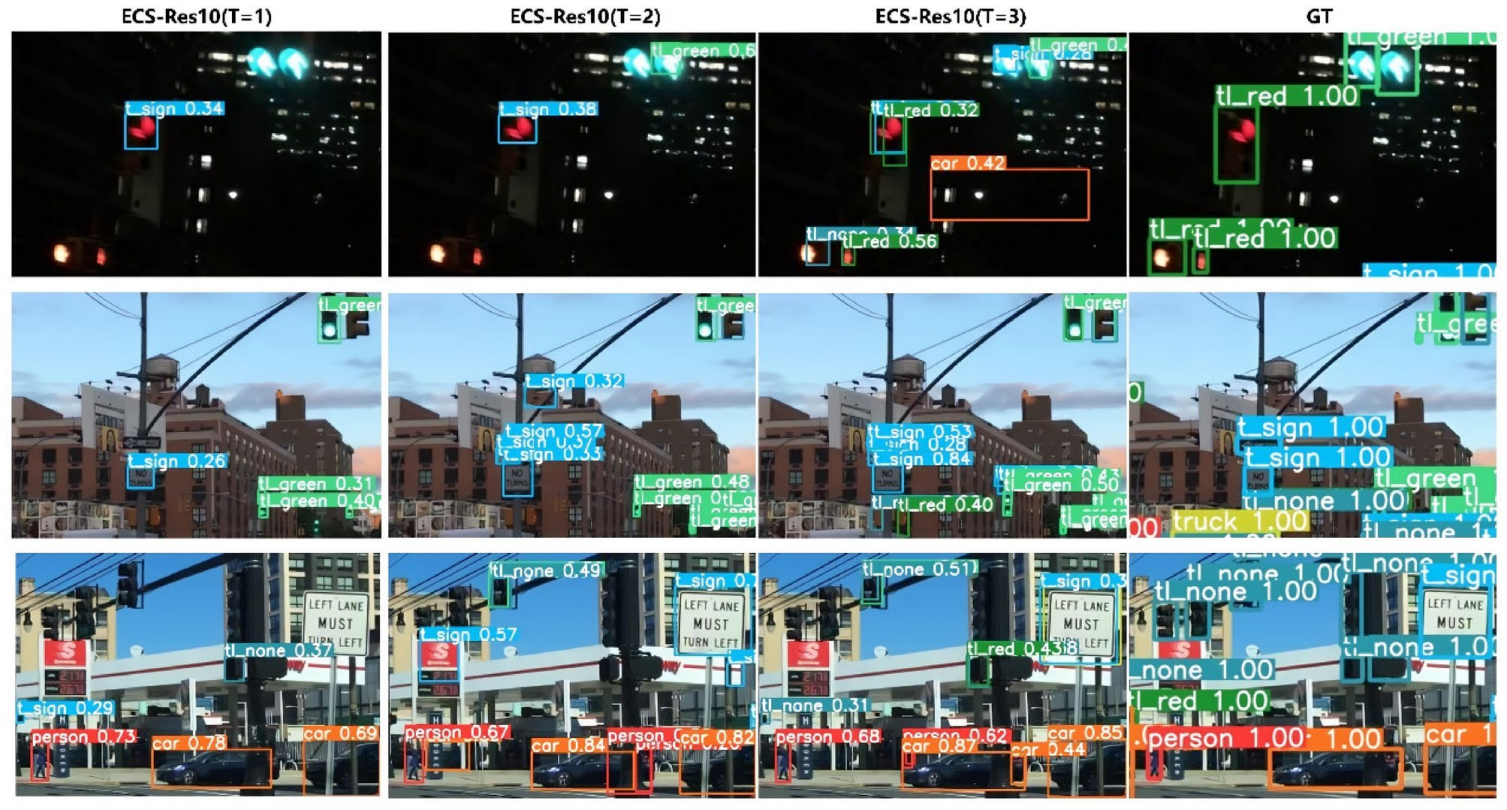
Detection results of ECS-YOLO (Res10) model on the BDD100K dataset in different time steps.

According to the data in Table 4, as the time step *T* of the spiking neural network increases, the accuracy and robustness of target detection significantly improve. This trend is visualized in Fig. 4, showing that longer time steps enhance the detection accuracy of the ECS-YOLO model, thereby improving its applicability in dynamic driving scenarios.

It is essential to acknowledge that the complete BDD100K dataset was not utilized for testing due to constraints associated with the experimental equipment, which consequently led to relatively low absolute values in the aforementioned results. Nonetheless, these preliminary findings adequately illustrate the trend regarding the impact of time steps on model performance. Our experimental outcomes highlight the significance of time steps and offer guidance for the further optimization of the ECSLIF-YOLO model.

## Conclusion

This paper presents ECSLIF-YOLO, a novel spiking object detection model that integrates the dynamics of brain ECS with the LIF neuron mechanism. The model demonstrates significant accuracy on the KITTI and BDD100K datasets, achieving a maximum mean Average Precision (mAP) of 0.917, which is comparable to artificial neural networks (ANNs), while simultaneously reducing theoretical energy consumption by over 90%. Furthermore, ECSLIF-YOLO exhibits improved robustness in noisy environments, thereby establishing its importance for future intelligent transportation systems.

However, despite its impressive performance, challenges related to hardware adaptation remain due to the intricate spatio-temporal interactions inherent in the ECS-LIF mechanism, which complicate implementation and increase associated costs. Effective practical deployment necessitates a careful balance between computational resources and power limitations.

To facilitate the practical application of ECSLIF-YOLO, it is crucial to select efficient hardware architectures, such as Intel Loihi or IBM TrueNorth, to optimize latency, energy consumption, and accuracy. The stability of performance should be assessed through a three-stage evaluation process encompassing simulated environments, controlled test fields, and real-world road conditions. This comprehensive approach ensures dependable operation in dynamic driving scenarios, thereby enhancing the prospects for successful integration into real-world applications.

## Data Availability

The data supporting this study’s findings are available from the corresponding author upon reasonable request.
